# Prescription Opioid Misuse Among High School Athletes During the Opioid Prescribing Limit Law Era: YRBSS 2017–2023

**DOI:** 10.3390/healthcare14152407

**Published:** 2026-08-05

**Authors:** Francine R. Vega, Andrea J. Yatsco, Audrey Sarah Cohen, James R. Langabeer, Tiffany Champagne-Langabeer, Rakshitha Vijendra, Christine Bakos-Block

**Affiliations:** Center for Behavioral Emergency and Addiction Research, The University of Texas Health Science Center, Houston, TX 77030, USA; andrea.j.yatsco@uth.tmc.edu (A.J.Y.); james.r.langabeer@uth.tmc.edu (J.R.L.); tiffany.champagne@uth.tmc.edu (T.C.-L.); christine.bakosblock@uth.tmc.edu (C.B.-B.)

**Keywords:** adolescent opioid misuse, sports participation, concussion, opioid prescribing limit laws, health disparities, Youth Risk Behavior Surveillance System

## Abstract

**Highlights:**

**What are the main findings?**

- National trends during the opioid prescribing limit law implementation era did not fully capture important subgroup differences in prescription opioid misuse.
- Hispanic/Latino and Black/African American students demonstrated significant concussion-related associations with prescription opioid misuse while not experiencing the significant temporal decline observed among White students.

**What are the implications of the main findings?**

- Population-level trends should be complemented by subgroup analyses to identify students at the highest risk for prescription opioid misuse.
- The persistence of elevated risk among selected subgroups suggests that prescribing policies alone may be insufficient to address adolescent prescription opioid misuse. Concussion management protocols for school athletes and culturally responsive, community-based prevention strategies should complement broader prescribing policies.

**Abstract:**

**Background/Objectives:** School-age athletes occupy a paradoxical position in the adolescent opioid crisis, combining health-oriented activity with elevated risk of sports-related injury and prescription opioid exposure. In response to the opioid epidemic, states enacted opioid prescribing limit laws (OPLLs) beginning in 2016–2017, yet whether these supply-side policies reached high-risk youth subgroups remains unclear. This study evaluated national temporal trends in prescription opioid misuse among U.S. high school students during the period of OPLL implementation and examined differences across athletic participation, concussion history, sex, grade, race/ethnicity, and substance-use profiles. **Methods:** Using four biennial waves of the Youth Risk Behavior Surveillance System with a consistent prescription pain-medicine measure (2017, 2019, 2021, and 2023; total N = 44,501), we conducted survey-weighted multivariable logistic regression models producing adjusted odds ratios (aORs) for lifetime prescription opioid misuse, controlling for survey year, sports participation, sex, grade, race/ethnicity, alcohol use, and other illicit drug use. Interaction and stratified models examined differences associated with sports participation, concussion history, demographic characteristics, and substance use. **Results:** Weighted prescription opioid misuse declined from 13.9% in 2017 to 12.0% in 2023, with a significant unadjusted linear decline per two-year survey wave (OR = 0.936; 95% CI: 0.879–0.997; *p* = 0.040). This overall trend was attenuated after adjustment (aOR = 0.967; 95% CI: 0.914–1.024; *p* = 0.248). Temporal patterns differed significantly across race/ethnicity, sex, grade, and selected substance-use groups. White and male students demonstrated significant adjusted linear declines, whereas Hispanic/Latino and female students remained flat. Black/African American students showed significant year-to-year variation, with elevated adjusted odds in 2019 and 2021 relative to 2017. Concussion history was independently associated with misuse among athletes (aOR = 1.645; 95% CI: 1.417–1.909; *p* < 0.001), with significant associations observed among White, Asian, Black/African American, and Hispanic/Latino athletes. The pooled concussion-by-race/ethnicity interaction, however, was not significant (*p* = 0.295). **Conclusions:** Although prescription opioid misuse declined descriptively between 2017 and 2023, adjusted analyses did not identify a significant overall national temporal change. Since subgroup patterns diverged, prevention planning and surveillance should be disaggregated by race/ethnicity and sex rather than guided by national totals, and groups whose misuse did not decline may require strategies extending beyond clinical prescribing controls.

## 1. Introduction

School-age athletes occupy a paradoxical position in the adolescent opioid crisis: health-oriented and physically active, yet at elevated risk of sports-related injury and subsequent exposure to prescription opioids. Sports participation and concussion history have been identified as significant predictors of opioid misuse among U.S. adolescents. Concussion may increase vulnerability to prescription opioid misuse through injury-related pain, potential analgesic exposure, and persistent post-concussive symptoms [1,2]. Concussion symptoms may include headache and other pain [3], sleep disruption [4], and depressive symptoms [5,6]. Each is independently associated with substance use in adolescence. Adolescent chronic pain independently predicted prescription opioid misuse in adulthood in a national longitudinal cohort [7], and adolescent sleep deficiency was prospectively associated with the same outcome after adjustment for chronic pain, mental health, and prior substance use [8]. Cho et al. followed 3204 baseline never-users of nonmedical prescription opioids across 42 months and found that major depression at baseline predicted subsequent nonmedical prescription opioid use. Concussion may therefore raise misuse risk indirectly by producing the conditions under which adolescents could self-medicate.

A further pathway is behavioral rather than injury-specific. In a national sample of 25,408 eighth-, tenth-, and twelfth-grade students, adolescents reporting multiple diagnosed concussions had roughly twice the odds of recent substance use across every category examined, including nonmedical prescription drug use, after adjustment for potential confounders [9]. This vulnerability sits within a broader epidemic. The misuse of opioids among children and adolescents has emerged as a critical public health crisis, driven not only by prescription medications but also by illicit and manufactured drugs such as heroin and fentanyl. For the first time, drug overdose has become the leading cause of unintentional death in the United States, surpassing motor vehicle accidents and firearms [10]. Among youth, opioid misuse serves as a key precursor to adverse outcomes, including opioid use disorder and overdose [11]. A substantial portion of opioid-related deaths among children have been linked to prescription opioids, with 73% of such deaths in 2019 involving prescribed medications [12,13].

Over the past two to three decades, healthcare providers have significantly increased opioid prescribing to youth (adolescents aged 12–17 years and young adults aged 18–24 years) in the United States [14]. This rise has been attributed in part to early perceptions that opioids were safe. Pharmaceutical marketing that minimized addiction risks reinforced these perceptions [15,16,17]. Recent studies document declining pediatric opioid prescribing, including reductions following minor orthopedic injuries and broader national declines in opioid dispensing among children and adolescents through 2022 [18,19]. Despite the decline in prescribing rates, opioids remain a significant cause of mortality for this age group.

Additionally, opioid-prescribing patterns differ across racial and ethnic groups, with non-Hispanic White youth more likely to receive opioid prescriptions than non-Hispanic Black or Hispanic/Latino youth [19,20]. These differences may also reflect structural factors, including unequal access to pediatric and sports medicine services, language and communication barriers, differences in insurance coverage, and disparities in access to athletic trainers and post-injury follow-up care [19,20]. While these factors may shape how and where adolescents encounter prescription opioids, they may also contribute to differences in misuse across racial and ethnic groups.

Recent American Academy of Pediatrics guidance recommends a multimodal approach to acute pain management in children and adolescents, combining nonpharmacologic therapies and non-opioid medications and reserving opioids for situations in which they are clinically indicated [21]. Legitimate reasons for prescribing opioids for youth include major surgical procedures, major trauma such as vehicular accidents, cancer, and sickle cell anemia. In question is the use of opioid prescriptions to treat pain from headaches, minor surgical procedures, sports injuries, and dental procedures [22]. Miech et al. [23] reported adolescents with legitimate prescription opioids prior to 12th grade were 33% more likely to report opioid misuse following high school, particularly among low-risk individuals with little prior exposure to other substances. For drug-naïve youth, an opioid prescription often serves as a first encounter with an addictive substance, and the perceived safety of a medically sanctioned prescription may lower barriers to subsequent misuse [23,24,25]. The likelihood of long-term opioid use has been tied to initial prescribing decisions. Longer days’ supply on the first prescription and additional opioid dispensing during the first episode of care both increased this risk. Unfortunately, much of the evidence linking opioid prescribing to long-term opioid use is derived from adult populations. How prescribing practices shape opioid-related behaviors among children and adolescents is far less understood [14].

In response to the national opioid crisis, states began enacting opioid prescribing limit laws (OPLLs) beginning around 2016–2017, targeting clinical prescribing practices and limiting supply-side access to prescription opioids. Of the 39 states that have adopted a prescribing limit law, only 16 include provisions specific to minors [26,27,28]. Whether these laws have reached high-risk youth subgroups, athletes, youth with concussion histories, and underserved adolescents remains poorly understood. In particular, there is a lack of nationally representative, longitudinal evidence assessing how opioid use trends among adolescent student-athletes have evolved in the context of OPLL implementation. Addressing this gap is essential to determining whether current policy approaches effectively reduce misuse among vulnerable youth populations or leave important disparities unaddressed.

The present study extends prior research by examining national trends through 2023 using a consistent prescription pain-medicine measure; evaluating whether temporal patterns differed by sports participation, concussion history, sex, grade, and race/ethnicity; estimating race-stratified concussion associations among athletes; and examining whether trends varied across substance-use profiles. Therefore, the objective of this study is to evaluate temporal trends in prescription opioid misuse among U.S. high school students from 2017–2023 and to assess whether the implementation of opioid prescribing limit laws is associated with changes in use patterns across key subgroups. We hypothesize that use will decline following OPLL implementation overall, but that reductions will be smaller among high-risk groups and may vary across racial and ethnic populations.

## 2. Materials and Methods

### 2.1. Data Source and Study Design

This study used a repeated cross-sectional design with secondary data from four biennial waves of the Youth Risk Behavior Surveillance System (YRBSS): 2017, 2019, 2021, and 2023. The YRBSS is the leading public health surveillance system conducted by the Center for Disease Control and Prevention (CDC), monitoring health-risk behaviors among U.S. high school students in public and private schools. The YRBSS provides nationally representative data on substance use, sports participation, and injury history, enabling examination of these relationships across demographic subgroups and over time. This study leverages four waves of YRBSS data (2017–2023) to examine national trends in prescription opioid misuse among high school students in grades 9 through 12, with particular attention to the moderating roles of sports participation, concussion history, sex, and race/ethnicity in the context of state opioid prescribing limit laws (OPLL) implementation. The survey employs a three-stage cluster sampling design with stratification by metropolitan statistical area status and racial/ethnic concentration, yielding a nationally representative sample of students in public and private schools across all 50 states in the USA including the District of Columbia. Puerto Rico, U.S. territories, and the Virgin Islands were excluded from the national sampling frame [29].

The primary analytic dataset included four survey waves (2017, 2019, 2021, 2023; N = 44,501), spanning a period of expanding enactment and implementation of OPLLs with 2017 serving as the reference year. Earlier waves were excluded because, in 2015 and earlier, the questionnaire assessed misuse of a broader category of prescription drugs that included opioids, stimulants, and benzodiazepines, whereas beginning in 2017, the YRBSS specifically assessed prescription pain-medication misuse.

### 2.2. Outcome Variable

The primary outcome was lifetime prescription opioid misuse, operationalized using the YRBSS prescription pain-medicine misuse item, consistent with prior YRBSS research [2]. Students were asked how many times during their life they had taken prescription pain medicine without a doctor’s prescription or differently from how a doctor instructed them to use it. Responses were dichotomized as no misuse (0 times; coded 0) or any lifetime misuse (one or more times; coded 1). The lifetime measure was selected because it was consistently available across all four study waves; the past-30-day item was not available until 2019.

### 2.3. Primary Exposures and Moderators

Sports participation was defined as playing on one or more school or community sports teams in the past 12 months (QN80; 1 = Yes, 0 = No). Concussion history was defined as having sustained at least one concussion or head injury from playing a sport or being physically active (QN81; 1 = Yes, 0 = No). For the primary temporal analyses, survey year was modeled categorically, with 2017 as the reference year.

### 2.4. Covariates

Demographic covariates included sex (female/male with female being the reference variable), grade as a proxy for age (9th, 10th, 11th, 12th with 9th being the reference variable), and race/ethnicity. Consistent with federal standards for Hispanic ethnicity classification, students who identified as Hispanic or Latino were classified as Hispanic/Latino (any) regardless of their racial identification. Students classified as Hispanic/Latino (any), however, are not a homogeneous group. Prior analyses of Youth Risk Behavior Survey data have reported differences in behavioral health outcomes between multiracial Hispanic and single-race Hispanic adolescents [30]. Racial self-identification is also not fixed: adolescents asked nearly identical race questions within the same study do not always answer them the same way [31], and race and Hispanic-origin responses change when the same individuals are surveyed again years later, most often among those reporting multiple races [32]. Following federal classification standards, the two groups were therefore combined, Hispanic/Latino (raceeth = 6) and Multiple Hispanic (raceeth = 7), into a single group. Estimates for Hispanic/Latino students should be interpreted as an average across a heterogeneous population. The remaining categories were retained as: American Indian/Alaska Native, Asian, Black/African American, Native Hawaiian/Pacific Islander, White (reference variable), and Multiple Non-Hispanic.

Substance-use measures included alcohol use, binge drinking, marijuana use, and other illicit drug use, each coded as binary (1 = Yes, 0 = No). Other illicit drug use was defined as reported use of any of the following substances: cocaine, inhalants, heroin, methamphetamine, or ecstasy. Alcohol use and other illicit drug use were included as adjustment variables in the primary models. Alcohol use, binge drinking, marijuana use, and the individual illicit substances were evaluated separately as moderators of temporal trend score.

### 2.5. Statistical Analysis

All analyses were conducted in R (version 4.5.0) using the survey package version 4.4–8 to account for the complex cluster sampling design. Survey weights, primary sampling units (PSU), and strata were specified in all models using the svydesign() function with nest = TRUE. Because the public-use national YRBSS files do not include school identifiers, clustering was addressed using the provided PSU, stratum, and weight variables; no additional school-level clustering term was specified. Weighted prevalence estimates were calculated using svymean() and svyby(), and categorical comparisons used Rao–Scott-corrected chi-squared tests.

Analyses used a complete-case approach, with no imputation of missing values. Of the 44,501 respondents in the four-wave dataset, 40,182 (90.3%) had complete data for the primary regression variables and were included in the adjusted models; 4319 respondents (9.7%) were excluded because of missing data on one or more model variables. Generalized variance inflation factors were examined to assess multicollinearity among the covariates in the primary adjusted model. Survey-weighted logistic regression models were estimated using svyglm() with a quasibinomial family and logit link. Model 1 estimated the unadjusted association between sports participation and opioid misuse. Model 2 included categorical survey year, sports participation, alcohol use, other illicit drug use, sex, grade, and race/ethnicity. Model 3 added the sports participation-by-survey-year interaction. Supporting models treated survey wave as an ordinal two-year measure to estimate linear temporal trends and the sports participation-by-wave interaction. Model 4 was restricted to athletes with complete concussion data (N = 20,069) and examined the association between concussion history and opioid misuse while adjusting for categorical survey year and all covariates. Results are reported as odds ratios (ORs) with 95% confidence intervals. Separate interaction models evaluated whether categorical survey-year patterns differed by race/ethnicity, sex, grade, concussion history, alcohol use, and other illicit drug use. Corresponding linear interaction models evaluated whether the direction or magnitude of change per two-year survey wave differed across these groups. Concussion-by-year models were restricted to athletes. Design-based joint Wald tests were used to evaluate categorical-year and interaction terms. When an interaction was significant, subgroup-specific categorical-year estimates and adjusted linear trends were examined to characterize the pattern. Because six moderator families were evaluated, Bonferroni- and false-discovery-rate-adjusted *p*-values were calculated for the omnibus interaction tests. Statistical significance was assessed at α = 0.05. Interaction terms used reference-cell coding and were not mean-centered. Binary moderators were coded 0/1, categorical variables used the reference groups specified above, and the linear survey-wave variable was coded 0, 1, 2, and 3, with 2017 anchored at zero.

## 3. Results

### 3.1. Sample Characteristics

The primary analytic sample included n = 44,501 students across four survey waves: n = 11,670 in 2017, n = 9735 in 2019, n = 10,340 in 2021, and n = 12,756 in 2023. The sample was approximately evenly distributed by sex, with female students comprising n = 22,049 (49.2%) and male students comprising n = 22,071 (50.8%). Students were nearly evenly distributed across grades, ranging from 26.5% in ninth grade (n = 11,646) to 23.6% in twelfth grade (n = 10,098). White students comprised the largest group (n = 20,253; 50.2%), followed by Hispanic/Latino students (n = 10,433; 26.7%), Black/African American students (n = 6172; 11.9%), Multiple Non-Hispanic students (n = 3083; 5.7%), Asian students (n = 2156; 4.4%), and with American Indian/Alaska Native and Native Hawaiian/Pacific Islander students each comprising 0.5% (n = 278, and n = 1250 respectively). Overall, 53% (n = 23,629) students reported participating in one or more sports teams, 13.8% reported a concussion history, and 13.0% (n = 5970) students reported prescription opioid misuse. Unweighted counts and weighted prevalence estimates, along with opioid misuse rates by subgroup, are presented in Table 1.

### 3.2. Overall Trends in Opioid Misuse and Substance Use

Every substance examined was substantially more prevalent among students who reported prescription opioid misuse than among those who did not. Alcohol use was reported by 51.1% of opioid misusers (n = 2694), compared with 22.3% of non-misusers (n = 7908). Marijuana use was reported by 41.1% of opioid misusers (n = 2428), compared with 15.2% of non-misusers (n = 6202). Illicit substances showed similarly pronounced differences: cocaine use (16.6%, n = 957 vs. 1.3%, n = 504), inhalant use (22.6%, n = 1332 vs. 4.2%, n = 1595), methamphetamine use (10.2%, n = 597 vs. 0.7%, n = 261), ecstasy use (16.0%, n = 930 vs. 1.2%, n = 480), and heroin use (7.9%, n = 479 vs. 0.5%, n = 191; all Rao–Scott-corrected *p* < 0.001). American Indian/Alaska Native youth had the highest descriptive prevalence across all seven substance categories, although racial/ethnic differences in inhalant use were not statistically significant (*p* = 0.063); the groups with the lowest prevalence varied by substance. Females reported higher prevalence of alcohol, marijuana, and inhalant use, whereas males reported higher prevalence of cocaine, heroin, methamphetamine, and ecstasy use.

#### 3.2.1. National Time Trends in Prescription Opioid Misuse, 2017–2023

Table 2 presents the national time trends in prescription opioid misuse from 2017–2023. The weighted prevalence of prescription opioid misuse was 13.9% in 2017 (n = 1629), 13.9% in 2019 (n = 1447), 12.3% in 2021 (n = 1321), and 12.0% in 2023 (n = 1573). In the adjusted complete-case model (n = 40,182), survey year was not jointly associated with prescription opioid misuse (joint Wald *p* = 0.451). Relative to 2017, the adjusted odds of misuse did not differ significantly in 2019 (aOR = 1.000; 95% CI: 0.821–1.218; *p* = 0.998), 2021 (aOR = 0.995; 95% CI: 0.831–1.191; *p* = 0.953), or 2023 (aOR = 0.896; 95% CI: 0.748–1.073; *p* = 0.232). The supporting linear-trend model also showed no significant overall change per two-year survey wave (aOR = 0.967; 95% CI: 0.914–1.024; *p* = 0.248). Significant changes were observed in several related indicators across survey years. Alcohol use decreased from 29.9% in 2017 (n = 2970) to 22.3% in 2023 (n = 2762), with significant categorical and linear trends (both *p* < 0.001). Other illicit drug use decreased from 24.5% in 2017 (n = 2909) to 21.0% in 2023 (n = 2931), also with significant categorical and linear trends (both *p* < 0.001). Sports participation varied across survey years—54.3% in 2017 (n = 6387), 57.4% in 2019 (n = 5515), 49.1% in 2021 (n = 5103), and 52.4% in 2023 (n = 6624)—with a significant categorical-year test (*p* = 0.006) but no significant linear trend (OR = 0.948; 95% CI: 0.892–1.007; *p* = 0.082). The lower sports-participation estimate in 2021 should be interpreted in the context of pandemic-related disruptions to schooling and organized athletics. Concussion prevalence likewise varied significantly across individual survey years (categorical *p* < 0.001), ranging from 11.7% in 2021 (n = 1192) to 15.0% in 2017 (n = 1702), without evidence of a significant linear trend (OR = 0.953; 95% CI: 0.898–1.010; *p* = 0.104).

#### 3.2.2. Moderation by Concurrent Substance Use

Alcohol use, binge drinking, and marijuana use each significantly moderated national trends in prescription opioid misuse (interaction aOR = 0.806, 0.815, and 0.800, respectively; all *p* < 0.001; Table 3). For each of these three substances, the adjusted odds of opioid misuse declined significantly across survey waves among students reporting that behavior, whereas no significant trend was observed among students who did not. All three interactions remained significant after Bonferroni and false-discovery-rate correction.

**Figure 1:**
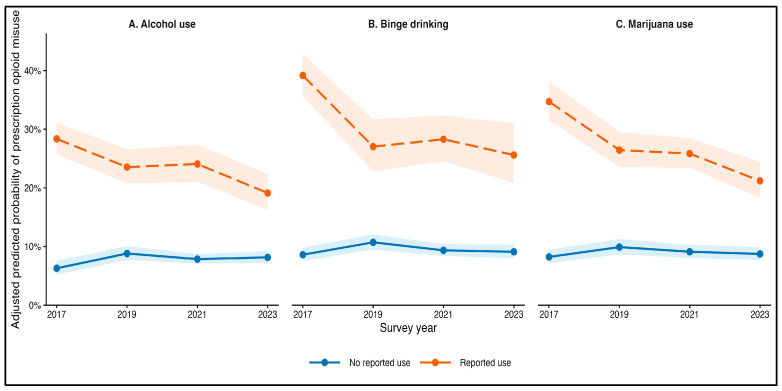
Adjusted predicted probabilities of prescription opioid misuse by alcohol use, binge drinking, and marijuana use across OPLL implementation years, 2017–2023. Note. Estimates were obtained from separate survey-weighted logistic regression models adjusted for sports participation, sex, grade, and race/ethnicity. Lines distinguish students who reported versus did not report each substance; shaded regions represent 95% confidence intervals.

Cocaine, inhalant, heroin, methamphetamine, and ecstasy use did not significantly moderate either the categorical or linear trends. Adjusted predicted probabilities are shown in Figure 1.

### 3.3. Logistic Regression Models

Results from the four primary models are presented in Table 4. Sports participation was not associated with opioid misuse in the unadjusted model (Model 1; n = 44,501; OR = 0.938; 95% CI: 0.857–1.025; *p* = 0.156) but was associated with modestly lower odds after adjustment (Model 2; n = 40,182; aOR = 0.900; 95% CI: 0.818–0.989; *p* = 0.029). No problematic multicollinearity was identified among the covariates (maximum adjusted GVIF = 1.216).

In the adjusted model, alcohol use and other illicit drug use were the strongest correlates of misuse (aOR = 2.170 and 3.691, respectively; both *p* < 0.001). Female students had higher adjusted odds of misuse than male students (male aOR = 0.758; 95% CI: 0.696–0.824; *p* < 0.001), and odds declined with grade, with significantly lower odds among eleventh- and twelfth-grade students than among ninth-grade students. Relative to White students, Hispanic/Latino students (aOR = 1.235; 95% CI: 1.086–1.404; *p* = 0.001) and Multiple Non-Hispanic students (aOR = 1.346; 95% CI: 1.108–1.635; *p* = 0.003) had higher adjusted odds of misuse; no other racial or ethnic group differed significantly.

The sports participation by survey-year interaction was not significant in either the categorical specification (Model 3; joint Wald *p* = 0.306) or the supporting linear specification (aOR = 1.005; 95% CI: 0.927–1.090; *p* = 0.896; Table S1), indicating that the association between sports participation and misuse did not change across survey years.

Model 4 was restricted to athletes with available concussion data (n = 20,069). Concussion history was independently associated with higher odds of prescription opioid misuse (aOR = 1.645; 95% CI: 1.417–1.909; *p* < 0.001). Race/ethnicity-stratified models among athletes demonstrated significant associations among White students (n = 9921; aOR = 1.578; 95% CI: 1.301–1.915; *p* < 0.001), Asian students (n = 899; aOR = 3.270; 95% CI: 1.374–7.779; *p* = 0.008), Black/African American students (n = 2714; aOR = 1.827; 95% CI: 1.132–2.948; *p* = 0.014), and Hispanic/Latino students (n = 4317; aOR = 1.834; 95% CI: 1.446–2.327; *p* < 0.001). Estimates for American Indian/Alaska Native, Multiple Non-Hispanic, and Native Hawaiian/Pacific Islander athletes were not statistically significant and were imprecise in the smaller subgroups. The overall concussion-by-race/ethnicity interaction was not significant (joint Wald *p* = 0.295), providing no evidence that the strength of the concussion association differed across racial and ethnic groups (Table S1).

### 3.4. Demographic and Concussion-Related Heterogeneity in National Trends During OPLL Implementation, 2017–2023

Although sex, grade, and race/ethnicity were associated with overall opioid-misuse odds in the primary models, additional interaction analyses examined whether national trends differed across subgroups during the broader period of OPLL implementation from 2017 through 2023 (Table 5). These analyses describe heterogeneity in temporal patterns during the implementation period.

Among complete cases (n = 40,182), categorical survey-year trends differed significantly by race/ethnicity (joint Wald *p* = 0.004), sex (*p* = 0.007), and grade (*p* < 0.001), and all three interactions remained significant after both Bonferroni and false-discovery-rate correction. The corresponding linear interactions were also significant (race/ethnicity *p* = 0.011; sex *p* = 0.021; grade *p* < 0.001), although only the grade interaction survived Bonferroni correction.

Subgroup-specific estimates indicated significant declines among White students (aOR = 0.917; 95% CI: 0.847–0.994; *p* = 0.034) and male students (aOR = 0.929; 95% CI: 0.870–0.993; *p* = 0.031), with lower adjusted odds in 2023 than in 2017 in both groups. No significant linear trend was observed among Hispanic/Latino students (aOR = 1.010; 95% CI: 0.932–1.095; *p* = 0.807) or female students (aOR = 1.001; 95% CI: 0.935–1.071; *p* = 0.983), and adjusted odds in 2023 were essentially unchanged from 2017 in both. Black/African American students showed no significant linear trend (aOR = 1.097; 95% CI: 0.977–1.232; *p* = 0.115), but were the only racial or ethnic group whose categorical year pattern varied significantly (joint Wald *p* = 0.008), with elevated adjusted odds in 2019 (aOR = 1.573; 95% CI: 1.128–2.194; *p* = 0.008) and 2021 (aOR = 1.635; 95% CI: 1.186–2.254; *p* = 0.003) relative to 2017.

Grade-specific patterns moved in opposing directions. Among ninth-grade students, adjusted odds increased across study years (aOR = 1.105; 95% CI: 1.021–1.197; *p* = 0.014) and were higher in 2023 than in 2017 (aOR = 1.348; 95% CI: 1.047–1.734; *p* = 0.021). Among twelfth-grade students, adjusted odds declined across years (aOR = 0.819; 95% CI: 0.743–0.902; *p* < 0.001) and were lower in 2023 than in 2017 (aOR = 0.525; 95% CI: 0.385–0.716; *p* < 0.001). No significant linear trends were observed among tenth- or eleventh-grade students. Among athletes, trends did not differ by concussion history in either the categorical-year (joint Wald *p* = 0.672) or linear-trend model (*p* = 0.562).

## 4. Discussion

### 4.1. Overview of Findings

This study examined national trends in prescription opioid misuse among U.S. high school students from 2017 to 2023, a period corresponding to widespread implementation of state opioid prescribing limit laws (OPLLs). Although weighted prevalence declined descriptively from 13.9% in 2017 to 12.0% in 2023, neither the adjusted categorical-year model nor the supporting linear-trend model demonstrated a significant overall national change. This indicates that prescription opioid misuse remained a persistent concern among adolescents during the OPLL implementation period. Concussion history emerged as a robust independent risk factor among athletes, including significant associations among White, Asian, Black/African American, and Hispanic/Latino athletes. The concussion-by-race/ethnicity interaction was not significant, indicating that the strength of this association did not differ statistically across racial and ethnic groups. However, racial and ethnic differences were evident in the temporal findings. White students demonstrated a significant linear decline in prescription opioid misuse during the OPLL implementation years, whereas Hispanic/Latino and Black/African American students did not experience comparable linear declines. Black/African American students instead demonstrated significant non-linear variation across survey years, with elevated adjusted odds in 2019 and 2021 relative to 2017.

Hispanic/Latino students demonstrated essentially no adjusted change in prescription opioid misuse during the OPLL implementation years. Both the linear trend estimate and the 2023-versus-2017 comparison were approximately null, contrasting with the significant decline observed among White students. This pattern suggests that the national reduction experienced by White youth was not shared by Hispanic/Latino youth.

Temporal patterns also diverged by grade, the only subgroup difference to survive Bonferroni correction in both specifications. Adjusted odds rose among ninth-grade students and fell by nearly half among twelfth-grade students, a difference between successive cohorts at each grade level rather than change as students progress through high school. The factors underlying this divergence were not examined in the present analysis.

### 4.2. Sports Participation and Concussion to Opioid Use Pathway

Sports participation was associated with modestly lower adjusted odds of prescription opioid misuse, and this association remained stable across survey years. Thus, the national relationship between sports participation and misuse did not change significantly during the OPLL implementation period. Athletic participation alone should therefore not be interpreted as a consistent indicator of increased prescription opioid misuse risk. Concussion history, however, was independently associated with higher odds of prescription opioid misuse among athletes. This finding is consistent with prior YRBSS research identifying sports-related concussion as a predictor of prescription opioid misuse among high school students [2]. Concussion may identify adolescents with greater exposure to injury, pain, healthcare encounters, sleep disruption, or psychological symptoms that increase vulnerability to substance misuse. Because the association persisted after adjustment for alcohol use, other illicit drug use, demographic characteristics, and survey year, concussion history appears to identify a clinically relevant subgroup of adolescent athletes.

Temporal trends did not differ significantly between athletes with and without concussion histories. This suggests that the elevated risk associated with concussion persisted throughout the OPLL implementation years rather than becoming substantially weaker over time. The mechanisms underlying this relationship are likely multifactorial. Directly, concussion and associated head trauma may be treated with prescription analgesics in acute care settings. Indirectly, concussion-related pain, sleep disruption, and psychological sequelae, including depression and anxiety, may increase vulnerability to substance misuse as a coping mechanism [11]. The persistent association observed here suggests that actual clinical practice or subsequent pain management may not fully align with current concussion management guidelines discouraging opioid analgesia, particularly in underserved communities. Although OPLLs may reduce the availability of opioids from some clinical sources, prescribing restrictions alone may not address the broader physical, behavioral, and psychosocial pathways linking injury history with misuse.

### 4.3. Race/Ethnicity, Concussion, and Prescription Opioid Misuse During OPLL Implementation

An important finding was the absence of a sustained linear decline in prescription opioid misuse among Hispanic/Latino and Black/African American students during the OPLL implementation years. Among Hispanic/Latino students, the linear trend indicated essentially no adjusted change across survey waves (aOR = 1.010; 95% CI: 0.932–1.095; *p* = 0.807), and adjusted odds in 2023 were nearly identical to those in 2017 (aOR = 1.016; 95% CI: 0.788–1.311; *p* = 0.901). Black/African American students also did not demonstrate a significant linear decline (aOR = 1.097; 95% CI: 0.977–1.232; *p* = 0.115), although their categorical pattern varied significantly, with elevated odds in 2019 and 2021. In contrast, White students demonstrated a significant linear decline. These findings indicate that improvements observed among White youth were not experienced uniformly across racial and ethnic groups during the broader OPLL implementation period. Differences in healthcare access, pain assessment and treatment, insurance coverage, communication, and clinical versus nonclinical sources of prescription opioids may contribute to these patterns [14,33,34]. However, these mechanisms were not directly measured and require further state-level and community-level investigation.

Concussion history was also associated with substantially higher odds of prescription opioid misuse among Hispanic/Latino athletes (aOR = 1.834; *p* < 0.001) and Black/African American athletes (aOR = 1.827; *p* = 0.014). Although the concussion-by-race/ethnicity interaction was not significant, these subgroup findings raise an important question about how injury management and access to follow-up care may shape opioid-misuse risk among adolescent athletes. Racial and ethnic disparities in pediatric pain management are well documented [19,20], and unequal access to sports-medicine infrastructure may represent an additional barrier. In a national analysis of 10,983 U.S. public secondary schools, schools with higher percentages of non-White students were more likely to lack athletic-trainer access, while schools with higher socioeconomic status generally had greater access to athletic-training services [35].

Because athletic trainers contribute to injury evaluation, concussion identification, care coordination, rehabilitation, and return-to-play monitoring, their absence may reduce opportunities for continuous school-based management following injury. Whether unequal access to these services contributes to the association between concussion and prescription opioid misuse remains an important question for future research.

### 4.4. Substance and Polysubstance Use Trends During OPLL Implementation

Alcohol use, binge drinking, and marijuana use each significantly moderated national trends in prescription opioid misuse during the OPLL implementation years. The substance-use-by-linear-survey-wave interactions were significant for alcohol use (aOR = 0.806; *p* < 0.001), binge drinking (aOR = 0.815; *p* < 0.001), and marijuana use (aOR = 0.800; *p* < 0.001). Among students reporting these behaviors, the adjusted odds of prescription opioid misuse declined across each two-year survey wave for alcohol use (aOR = 0.855; *p* < 0.001), binge drinking (aOR = 0.819; *p* < 0.001), and marijuana use (aOR = 0.807; *p* < 0.001). Comparable declines were not observed among students who did not report these behaviors. These interactions remained significant after both Bonferroni and false-discovery-rate corrections.

The results, however, should not be interpreted as suggesting that alcohol use, binge drinking, or marijuana use is protective. Across the study population, substance use remained strongly associated with prescription opioid misuse. Instead, the interaction findings indicate that the temporal pattern of opioid misuse differed according to students’ broader substance-use profiles. Students reporting these behaviors began the study period with substantially higher predicted probabilities of prescription opioid misuse and continued to represent high-risk groups despite subsequent declines.

By contrast, cocaine, inhalant, heroin, methamphetamine, and ecstasy use did not significantly moderate either the categorical or linear survey-year trends. Adolescents reporting these substances may experience more entrenched or complex polysubstance-use patterns that are less responsive to changes in clinical opioid availability. These interpretations were not tested but support other findings that adolescents may also obtain opioids through nonmedical channels that are less directly affected by clinical prescribing limits [33,34].

### 4.5. National Trends During OPLL Implementation

The national trends observed during 2017–2023 also unfolded alongside changes in prescribing guidance, monitoring, and public awareness. Although the 2016 CDC opioid-prescribing guideline was directed primarily toward adults with chronic pain rather than pediatric patients, its dissemination through clinician education, healthcare systems, payers, and prescribing tools may have contributed to broader changes in prescribing culture. The period also included increased use of prescription drug monitoring programs and state requirements for prescribers to review controlled-substance histories, together with growing clinical and public attention to opioid-related risks, safe storage, and disposal. These overlapping changes may have influenced prescription availability, prescribing practices, and perceptions of opioid safety. Their individual contributions cannot be separated in the present national trend analysis.

The lack of a significant adjusted national decline during 2017–2023 should not be interpreted as evidence that individual OPLLs were ineffective. State prescribing limits vary substantially in their timing, duration limits, dosage restrictions, exemptions, enforcement provisions, and inclusion of protections specifically addressing minors [26,28,36]. Nationally aggregated analyses combine students living under laws with markedly different designs and levels of implementation. Consequently, protective changes associated with stronger or more comprehensive laws may be diluted when combined with weaker statutes or laws lacking pediatric-specific provisions.

Prior evaluations have similarly reported limited or inconsistent associations between prescribing-cap laws and opioid prescribing among children and adolescents [27]. These findings may reflect both genuine limitations of supply-focused policies and the difficulty of representing heterogeneous state laws through a single national implementation period. The present results therefore characterize adolescent prescription opioid misuse during the broader years of OPLL implementation rather than the effect of a uniform national intervention.

The findings also support the need for state-level comparative analyses. Linking adolescent outcomes to the timing and specific provisions of state laws would permit examination of whether pediatric dosage limits, parental-consent requirements, education mandates, exemptions, or enforcement mechanisms are associated with different outcomes. Such analyses may identify effects that cannot be detected through national trend models. The design of such studies cannot determine the relative contribution of any single influence, and the pattern of results in this study is consistent with multiple concurrent forces. Interpreting the trends found in this study requires attention to a fundamental limitation in how OPLLs are typically assessed. The policy-evaluation literature has largely treated these laws as homogenous, modeling them as a binary indicator of whether a state has enacted a prescribing limit, despite substantial variation in their design, scope, and inclusion of minor-specific provisions [28,36]. This homogeneity assumption has consequences: rigorous quasi-experimental evaluations that pool laws of differing design have found no significant association between state prescribing limit laws and opioid prescribing among children and adolescents [27], or between limit laws and prescribing or overdose outcomes among adults [37]. These null findings may reflect not the ineffectiveness of prescribing restrictions per se, but the dilution that occurs when laws with and without pediatric-specific limits, consent requirements, and education mandates are analyzed as a single undifferentiated category.

The present analysis is subject to the same constraint. Because the YRBSS does not include state identifiers, students cannot be linked to the specific design of the law in effect in their state, and the observed national trend therefore reflects aggregate exposure to a heterogeneous mix of policies rather than to any single, well-specified intervention. Consistent with this interpretation, research pooling prescribing-cap laws have reported null associations with pediatric opioid prescribing and adult prescribing outcomes [27,37]. Whereas analyses distinguishing minor-specific law features have identified heterogeneous associations according to law design [28]. Collectively, this evidence suggests that pooled estimates may obscure differences between stronger, comprehensive laws and weaker statutes.

### 4.6. Limitations

Several limitations should be considered when interpreting these findings. The repeated cross-sectional design samples different students in each wave rather than following individuals over time. This structure supports inferences about population-level trends but not within-person change, and it precludes causal attribution. Observed associations may reflect secular trends, or concurrent interventions. Although the 2016 CDC opioid prescribing guidelines were primarily focused on adults, dissemination could have changed prescribing patterns and shifted social norms, which preceded formal state legislation in many jurisdictions. All variables were self-reported and are therefore subject to reporting and recall bias, particularly the underreporting of sensitive behaviors. Residual nonresponse bias may also remain despite survey weighting. Differential reporting across demographic subgroups may also result in outcome misclassification [38]. The YRBSS sampling frame also excludes out-of-school youth and dropouts, populations that may be at elevated risk of opioid misuse, potentially underestimating true prevalence. Because the national YRBSS sampling frame excludes U.S. territories, the findings may not be generalizable to adolescents residing in those jurisdictions.

A further limitation concerns the measurement of OPLL exposure. The public-use YRBSS does not include state identifiers, so respondents cannot be linked to the specific prescribing law in effect in their state. Exposure is therefore descriptive temporal patterns within the OPLL implementation period rather than as direct exposure to a defined statute. This approach inherits a broader limitation in the policy-evaluation literature, which has frequently modeled OPLLs as a homogenous binary, whether a state has enacted a prescribing limit, despite substantial variation in their comprehensiveness, scope, and inclusion of minor-specific provisions such as pediatric dosage caps, consent requirements, and education mandates [28,36]. Treating structurally dissimilar laws as a single category limits ability to estimate effects, as statutes with robust pediatric protections are averaged against those with none; in prior work, only comprehensive, minor-specific statutes showed protective associations with youth opioid mortality, while laws lacking pediatric provisions showed null or adverse associations [28]. This may partly explain why quasi-experimental evaluations pooling laws of differing design have detected no significant association between cap laws and pediatric opioid prescribing [27]. The present findings should therefore be interpreted as aggregate temporal trends across a heterogeneous policy environment rather than as the effect of any single intervention. Pooling structurally different OPLLs may obscure associations associated with particular law designs, including comprehensive, minor-specific provisions.

Several measurement constraints limit the scope of specific analyses. Before 2017, the YRBSS assessed misuse of a broader category of prescription medications that included stimulants, sedatives, and prescription pain medicines. Beginning in 2017, the YRBSS used a prescription pain medicine–specific item that remained comparable through 2023. The opioid-misuse analyses were therefore restricted to the 2017–2023 waves. The concussion item was not included in YRBSS until 2017, restricting concussion analyses to 2017–2023. Likewise, lifetime misuse was the only consistent outcome across all four waves; the prevalence estimates are expected to exceed those based on shorter recall periods, and the timing of misuse relative to concussion or the survey period cannot be determined. Race-stratified concussion estimates for American Indian/Alaska Native and Native Hawaiian/Pacific Islander athletes had wide confidence intervals because of small subgroup sample sizes and should be interpreted cautiously. In addition, the 2023 transition from paper-and-pencil to electronic tablet-based administration, together with supplemental sampling changes, may affect the comparability of the 2023 wave with earlier years [29]. As noted in Section 2.4, Hispanic/Latino students were analyzed as a single group. Estimates for this group therefore represent averages across multiracial and single-race Hispanic youth, and divergent patterns within the group could not be distinguished; therefore, these estimates should be read as group averages rather than as estimates for either component.

### 4.7. Implications for Policy and Practice

These findings carry several implications for public health policy and clinical practice. First, the lack of decline among female students versus the significant decline observed among male students supports the importance of continued attention to sex-specific patterns in opioid misuse.

Second, the absence of sustained linear declines among Hispanic/Latino and Black/African American youth, along with the significant concussion-related risk within both populations, supports the need for culturally responsive, community-level implementation strategies that extend beyond clinical prescribing restrictions. Outreach efforts should target school-based athletic programs, community health centers, and sports medicine providers serving diverse communities, with attention to language access, health literacy, access to athletic training and follow-up care, and non-opioid pain management education. Overall, however, concussion management protocols in high school athletics should explicitly address opioid prescribing practices, with emphasis on non-opioid analgesic alternatives and structured follow-up for students with concussion histories.

The polysubstance findings have direct implications for how prevention is targeted. Declines in prescription opioid misuse were concentrated among students who reported alcohol use, binge drinking, or marijuana use, whereas no significant decline was observed among students who did not report these behaviors. Because adolescents reporting other substance use are the group most readily identified by existing school-based screening and prevention programming, this pattern suggests that the students whose risk did not improve are precisely those least likely to be reached by substance-use-focused approaches. Prevention efforts that rely on alcohol or cannabis use as an indicator of broader risk may therefore under-identify adolescents who misuse prescription opioids in the absence of other substance use. These findings support universal rather than indicator-triggered screening for prescription opioid misuse, and they point to alternative identification pathways, including injury and concussion history, for adolescents who do not present with other substance-use behaviors.

Lastly, the subgroup-specific declines observed during the OPLL implementation period underscore the potential importance of well-designed, minor-specific prescribing laws; however, the present design cannot establish that the laws caused these changes or determine which law provisions may have contributed to them. Continued attention to prescribing practices in sports medicine and pediatric clinical settings remains warranted alongside complementary, non-supply-side strategies.

## 5. Conclusions

Prescription opioid misuse among U.S. high school students declined descriptively between 2017 and 2023; however, the adjusted analyses did not demonstrate a significant overall national trend, and patterns were not evenly distributed across groups. The study design cannot determine whether OPLLs contributed to the observed subgroup-specific changes; the pattern of results is consistent with multiple concurrent influences, including the 2016 CDC guideline, shifting prescribing norms, and changes in the illicit opioid supply.

Concussion history remained a significant risk factor for opioid misuse among adolescent athletes, including significant associations among Hispanic/Latino and Black/African American youth. In addition, Hispanic/Latino students did not experience sustained adjusted linear declines in misuse over time, highlighting persistent disparities that may not be addressed through supply-side prescribing policies alone. Adjusted odds of misuse also rose among ninth-grade students while declining among twelfth-grade students, the most robust subgroup difference observed. These findings support the need for comprehensive prevention strategies that combine responsible prescribing practices with improved injury management, non-opioid pain treatment, and early intervention, including culturally responsive interventions for high-risk youth. Sustaining progress in reducing adolescent opioid misuse will require a focused approach that addresses both clinical and social determinants of risk. Future research should use state-linked and longitudinal designs to examine specific OPLL provisions and distinguish policy-associated changes from broader secular trends.

## Figures and Tables

**Table 1:** Sample Characteristics and Prescription Opioid Misuse Prevalence by Survey Year.

Variable	2017 n = 11,670	2019 n = 9735	2021 n = 10,340	2023 n = 12,756	Total N = 44,501	Opioid Misuse n (%)	*p*-Value
***Outcomes***							
Opioid Misuse %	1629 (13.9%)	1447 (13.9%)	1321 (12.3%)	1573 (12.0%)	5970 (13.0%)	—	0.162
***Athlete Variables***							
Sports Participation	6387 (54.3%)	5515 (57.4%)	5103 (49.1%)	6624 (52.4%)	23,629 (53.0%)	3083 (12.6%)	0.156
Concussion History	1702 (15.0%)	1226 (14.6%)	1192 (11.7%)	1692 (14.0%)	5812 (13.8%)	1206 (20.8%)	<0.001
***Substance Use***							
Alcohol Use	2970 (29.9%)	2641 (29.8%)	2229 (23.0%)	2762 (22.3%)	10,602 (25.9%)	2694 (24.3%)	<0.001
Other Drug Use	2909 (24.5%)	2654 (26.4%)	2358 (21.4%)	2931 (21.0%)	10,852 (23.1%)	3254 (29.8%)	<0.001
***Sex***							
Female	5975 (51.2%)	4932 (49.6%)	4879 (48.0%)	6263 (48.1%)	22,049 (49.2%)	3327 (14.8%)	<0.001
Male	5606 (48.8%)	4719 (50.4%)	5313 (52.0%)	6433 (51.9%)	22,071 (50.8%)	2553 (11.0%)	
***Grade (Age Proxy)***							
9th (~14–15 yrs)	2995 (27.1%)	2591 (26.9%)	2685 (26.2%)	3375 (25.8%)	11,646 (26.5%)	1458 (12.4%)	0.532
10th (~15–16 yrs)	2915 (25.7%)	2683 (25.5%)	2555 (25.4%)	3372 (25.9%)	11,525 (25.6%)	1535 (12.7%)	
11th (~16–17 yrs)	2932 (24.2%)	2319 (23.9%)	2519 (24.7%)	3079 (24.2%)	10,849 (24.3%)	1493 (13.3%)	
12th (~17–18 yrs)	2730 (23.1%)	2063 (23.7%)	2492 (23.7%)	2813 (24.0%)	10,098 (23.6%)	1394 (13.1%)	
***Race/Ethnicity***							
Am Indian/Alaska Native	105 0.5%	92 0.6%	102 0.7%	951 0.3%	1250 (0.51%)	178 (20.6%)	<0.001
Asian	517 3.3%	454 4.7%	597 5.5%	588 4.2%	2156 (4.4%)	212 (9.9%)	
Black/African American	1946 11.9%	1377 10.8%	1561 11.9%	1288 12.8%	6172 (11.9%)	828 (12.6%)	
Native Hawaiian/PI	95 0.7%	39 0.3%	64 0.5%	80 0.5%	278 (0.5%)	30 (10.5%)	
White	5030 55.0%	4596 51.3%	4905 49.6%	5722 45.6%	20,253 (50.2%)	2501 (12.0%)	
Hispanic/Latino	3106 23.0%	2451 27.8%	2231 25.8%	2645 30.0%	10,433 (26.7%)	1566 (14.6%)	
Multiple Non-Hispanic	646 5.5%	463 4.5%	655 5.9%	1319 6.7%	3083 (5.7%)	500 (15.2%)	

Note. Percentages are survey-weighted; n values are unweighted counts. Opioid Misuse n (%) is the unweighted n with weighted subgroup prevalence. The across-year *p*-value uses a design-based joint Wald test; subgroup *p*-values use the Rao–Scott correction. Counts may not sum to the total because of item nonresponse. Hispanic/Latino includes Multiple Hispanic. The 2023 national YRBS included an American Indian/Alaska Native supplemental sample that intentionally oversampled AI/AN students to improve the precision of subgroup estimates.

**Table 2:** National Time Trends in Prescription Opioid Misuse and Related Indicators, 2017–2023.

Variable	2017 n (%)	2019 n (%)	2021 n (%)	2023 n (%)	Linear Trend OR/OR (95% CI)	Categorical *p*-Value	Linear Trend *p*-Value
***Unadjusted National Trends***							
Prescription Opioid Misuse	1629 (13.9%)	1447 (13.9%)	1321 (12.3%)	1573 (12.0%)	0.936 (0.879–0.997)	0.162	0.040
Sports Participation	6387 (54.3%)	5515 (57.4%)	5103 (49.1%)	6624 (52.4%)	0.948 (0.892–1.007)	0.006	0.082
Alcohol Use	2970 (29.9%)	2641 (29.8%)	2229 (23.0%)	2762 (22.3%)	0.858 (0.816–0.903)	<0.001	<0.001
Other Illicit Drug Use	2909 (24.5%)	2654 (26.4%)	2358 (21.4%)	2931 (21.0%)	0.919 (0.874–0.965)	<0.001	<0.001
Concussion History	1702 (15.0%)	1226 (14.6%)	1192 (11.7%)	1692 (14.0%)	0.953 (0.898–1.010)	<0.001	0.104

Note. Values for 2017–2023 are unweighted n (survey-weighted %). Each row represents a separate unadjusted survey-weighted logistic regression analysis. Linear-trend ORs represent change per two-year survey wave. Categorical-year *p*-values are design-based joint Wald tests evaluating differences across the four survey years. OR = odds ratio; CI = confidence interval; *p*-value considered significant at *p* < 0.05.

**Table 3:** Substance Use as a Moderator of National Trends in Prescription Opioid Misuse, 2017–2023.

Substance	Categorical Year Interaction *p*	Linear Interaction aOR	95% CI	*p*-Value	Non-Use Trend aOR (95% CI)	*p*-Value	Use Trend aOR (95% CI)	*p*-Value	Model N
Alcohol use	<0.001	0.806	0.744–0.872	<0.001	1.071 (1.000–1.147)	0.051	0.855 (0.795–0.921)	<0.001	40,190
Binge drinking	<0.001	0.815	0.740–0.898	<0.001	1.007 (0.946–1.072)	0.833	0.819 (0.747–0.898)	<0.001	39,462
Marijuana use	<0.001	0.800	0.736–0.869	<0.001	1.010 (0.948–1.076)	0.746	0.807 (0.750–0.869)	<0.001	42,737
Cocaine use	0.679	0.983	0.855–1.131	0.813	0.945 (0.894–0.999)	0.048	0.940 (0.821–1.076)	0.367	42,382
Inhalant use	0.754	0.998	0.895–1.112	0.966	0.897 (0.848–0.949)	<0.001	0.899 (0.809–1.000)	0.050	41,412
Heroin use	0.806	0.910	0.732–1.130	0.391	0.902 (0.856–0.950)	<0.001	0.818 (0.652–1.026)	0.082	42,528
Methamphetamine use	0.064	1.028	0.864–1.222	0.758	0.909 (0.863–0.958)	<0.001	0.950 (0.798–1.131)	0.561	42,509
Ecstasy use	0.358	0.949	0.813–1.107	0.501	0.920 (0.873–0.970)	0.002	0.887 (0.758–1.038)	0.136	42,120

Note. Each row represents a separate survey-weighted logistic regression model adjusted for sports participation, sex, grade, and race/ethnicity. The categorical-year *p*-value is the design-based joint Wald test for the substance × survey-year interaction. Linear interaction and subgroup trend aORs represent change per two-year survey wave; the linear interaction aOR compares the trend among those with substance use patterns with the trend among those without use. Alcohol use, binge drinking, and marijuana use remained significant moderators after both Bonferroni and false-discovery-rate correction across eight substances. N values are complete-case model sample sizes. aOR = adjusted odds ratio; CI = confidence interval. Interaction terms are multiplicative on the odds scale; the linear interaction aOR is the ratio of the per-wave odds ratio among students reporting the indexed substance to the per-wave odds ratio among students not reporting that substance, *p*-value significance at <0.05.

**Table 4:** Survey-Weighted Logistic Regression Models of Prescription Opioid Misuse, YRBSS 2017–2023.

Variable	Model 1 Unadjusted	Model 2 Adjusted	Model 3 Sports × Year	Model 4 Athletes Only
***Primary Predictors***				
Sports Participation (Ref: No)	0.938 (0.857–1.025) *p* = 0.156	0.900 * (0.818–0.989) *p* = 0.029	0.971 (0.830–1.135) *p* = 0.706	—
Survey Year (Ref: 2017)				
2019	—	1.000 (0.821–1.218) *p* = 0.998	1.139 (0.875–1.481) *p* = 0.331	0.889 (0.731–1.081) *p* = 0.236
2021	—	0.995 (0.831–1.191) *p* = 0.953	1.025 (0.818–1.286) *p* = 0.827	0.990 (0.811–1.209) *p* = 0.924
2023	—	0.896 (0.748–1.073) *p* = 0.232	0.914 (0.724–1.153) *p* = 0.445	0.882 (0.718–1.082) *p* = 0.227
***Sports Participation × Survey Year***				
Sports × 2019	—	—	0.789 (0.612–1.016) *p* = 0.066	—
Sports × 2021	—	—	0.946 (0.747–1.198) *p* = 0.644	—
Sports × 2023	—	—	0.966 (0.747–1.248) *p* = 0.789	—
Concussion History (Ref: No)	—	—	—	1.645 *** (1.417–1.909) *p* < 0.001
Alcohol Use (Ref: No)	—	2.170 *** (1.959–2.403) *p* < 0.001	2.170 *** (1.960–2.402) *p* < 0.001	2.126 *** (1.848–2.446) *p* < 0.001
Other Illicit Drug Use (Ref: No)	—	3.691 *** (3.348–4.069) *p* < 0.001	3.696 *** (3.353–4.073) *p* < 0.001	3.142 *** (2.727–3.621) *p* < 0.001
Male (Ref: Female)	—	0.758 *** (0.696–0.824) *p* < 0.001	0.757 *** (0.696–0.824) *p* < 0.001	0.711 *** (0.628–0.806) *p* < 0.001
***Grade (Ref: 9th)***				
10th Grade	—	0.933 (0.831–1.047) *p* = 0.236	0.932 (0.831–1.047) *p* = 0.234	0.913 (0.781–1.068) *p* = 0.255
11th Grade	—	0.869 * (0.770–0.981) *p* = 0.024	0.870 * (0.771–0.982) *p* = 0.024	0.834 * (0.705–0.986) *p* = 0.034
12th Grade	—	0.741 *** (0.652–0.841) *p* < 0.001	0.739 *** (0.651–0.840) *p* < 0.001	0.632 *** (0.524–0.763) *p* < 0.001
***Race/Ethnicity*** ***(Ref: White)***				
American Indian/Alaska Native	—	1.428 (0.934–2.183) *p* = 0.099	1.425 (0.934–2.176) *p* = 0.100	1.421 (0.784–2.573) *p* = 0.245
Asian	—	1.146 (0.920–1.426) *p* = 0.223	1.141 (0.915–1.422) *p* = 0.240	1.206 (0.859–1.693) *p* = 0.278
Black/African American	—	1.103 (0.930–1.308) *p* = 0.260	1.100 (0.928–1.304) *p* = 0.271	1.236 (0.981–1.557) *p* = 0.073
Hispanic/Latino (any)	—	1.235 ** (1.086–1.404) *p* = 0.001	1.232 ** (1.084–1.400) *p* = 0.001	1.368 *** (1.176–1.591) *p* < 0.001
Multiple Non-Hispanic	—	1.346 ** (1.108–1.635) *p* = 0.003	1.342 ** (1.105–1.629) *p* = 0.003	1.563 *** (1.238–1.972) *p* < 0.001
Native Hawaiian/Pacific Islander	—	1.029 (0.534–1.986) *p* = 0.931	1.026 (0.530–1.986) *p* = 0.940	0.547 (0.239–1.253) *p* = 0.153
Observations	44,501	40,182	40,182	20,069

Note. Results are OR or aOR (95% CI), followed by *p*-value. Model 1 estimates the unadjusted association between sports participation and opioid misuse. Model 2 adjusts for categorical survey year, sports participation, alcohol use, other illicit drug use, sex, grade, and race/ethnicity. Model 3 adds sports participation × categorical survey-year interaction terms. Model 4 is restricted to athletes with available concussion data and adjusts for categorical survey year and all covariates. The joint Wald *p*-values were 0.451 for survey year in Model 2 and 0.306 for the sports participation × survey-year interaction in Model 3. OR = odds ratio; aOR = adjusted odds ratio; CI = confidence interval. *** *p* < 0.001; ** *p* < 0.01; * *p* < 0.05; — = not included in model.

**Table 5:** Demographic and Concussion-Related Heterogeneity in National Trends in Prescription Opioid Misuse During OPLL Implementation, 2017–2023.

Characteristic	Categorical Interaction *p*-Value	Bonferroni/FDR *p*-Values	Linear Interaction *p*-Value	Bonferroni/FDR *p*-Values	Model N
Race/ethnicity	0.004	0.025/0.006	0.011	0.068/0.017	40,182
Sex	0.007	0.042/0.008	0.021	0.128/0.026	40,182
Grade	<0.001	<0.001/<0.001	<0.001	<0.001/<0.001	40,182
Concussion history	0.672	1.000/0.672	0.562	1.000/0.562	20,069
Panel B. Subgroup-Specific Adjusted Trends					
**Characteristic/Subgroup**	**Linear Trend aOR** ** (95% CI)**	***p*** **-Value**	**2023 vs. 2017 aOR** ** (95% CI)**	***p*** **-Value**	**Model N**
Race/ethnicity: White	0.917 (0.847–0.994)	0.034	0.760 (0.591–0.978)	0.033	18,952
American Indian/Alaska Native	0.737 (0.528–1.028)	0.072	0.577 (0.243–1.369)	0.210	1163
Asian	1.100 (0.906–1.335)	0.332	1.266 (0.670–2.390)	0.466	2036
Black/African American	1.097 (0.977–1.232)	0.115	1.399 (0.964–2.030)	0.077	5509
Hispanic/Latino (any)	1.010 (0.932–1.095)	0.807	1.016 (0.788–1.311)	0.901	9371
Multiple Non-Hispanic	0.896 (0.764–1.051)	0.176	0.789 (0.476–1.308)	0.357	2908
Native Hawaiian/Pacific Islander	0.901 (0.505–1.607)	0.720	0.820 (0.189–3.556)	0.788	243
Sex: Female	1.001 (0.935–1.071)	0.983	0.992 (0.801–1.229)	0.941	20,224
Male	0.929 (0.870–0.993)	0.031	0.794 (0.647–0.974)	0.027	19,958
Grade: 9th	1.105 (1.021–1.197)	0.014	1.348 (1.047–1.734)	0.021	10,560
10th	1.043 (0.960–1.133)	0.322	1.128 (0.875–1.455)	0.350	10,468
11th	0.921 (0.844–1.005)	0.065	0.800 (0.607–1.054)	0.112	9888
12th	0.819 (0.743–0.902)	<0.001	0.525 (0.385–0.716)	<0.001	9266
Concussion history: No	0.961 (0.889–1.039)	0.318	0.846 (0.661–1.083)	0.183	16,319
Yes	0.994 (0.893–1.106)	0.909	0.955 (0.685–1.333)	0.787	3750

Note. Panel A reports design-based joint Wald tests for characteristic × survey-year interactions. Multiplicity-adjusted *p*-values were calculated across all six prespecified moderators (race/ethnicity, sex, grade, concussion history, alcohol use, and other illicit drug use) separately for categorical-year and linear-trend specifications; only demographic and concussion results are shown here. Panel B reports subgroup-specific adjusted odds ratios. Linear-trend aORs represent change per two-year survey wave; categorical comparisons use 2017 as the reference. Race/ethnicity, sex, and grade models included n = 40,182 complete cases and adjusted for sports participation, alcohol use, other illicit drug use, sex, grade, and race/ethnicity, excluding the variable used to define each subgroup. Concussion analyses were restricted to athletes with available concussion data (n = 20,069) and adjusted for the same applicable covariates. These analyses characterize national temporal heterogeneity during the broader OPLL implementation period and do not estimate effects of individual state laws. aOR = adjusted odds ratio; CI = confidence interval; FDR = false discovery rate; OPLL = opioid prescribing limit law.

## Data Availability

The YRBSS data presented in this study were derived from the following resources available in the public domain: https://www.cdc.gov/yrbs/data/index.html, (accessed on 2 March 2026). The legal dataset (OPLLs) is currently being used in an ongoing study and is not yet publicly available.

## Supplementary Materials

The following supporting information can be downloaded at: https://www.mdpi.com/article/10.3390/healthcare14152407/s1, Table S1: Survey-Weighted Linear Trend and Sports Participation × Survey-Wave Interaction Models of Prescription Opioid Misuse, YRBSS 2017–2023.

## Abbreviations

The following abbreviations are used in this manuscript:

OPLL	Opioid Prescription Limit Laws
YRBSS	Youth Risk Behavior Surveillance System
CDC	Center for Disease Control and Prevention
