# Supplementary material for: Prescription Opioid Misuse Among High School Athletes During the Opioid Prescribing Limit Law Era: YRBSS 2017–2023
Source: Healthcare (Basel). 2026 Aug 5;14(15):2407. doi: 10.3390/healthcare14152407 (PMC13466084; doi:10.3390/healthcare14152407)
Supplement: Supplementary file 1 [file healthcare-14-02407-s001.zip › healthcare-4416583-supplementary.pdf]

Table S1. Survey-Weighted Linear Trend and Sports Participation × Survey-Wave Interaction Models of Prescription Opioid Misuse, YRBSS 2017–2023

Variable	Adjusted Linear-Trend Model	Sports × Linear-Trend Model
	Primary Predictors	
Sports Participation (Ref: No)	0.899* (0.818–0.987) p=0.026	0.892 (0.773–1.028) p=0.115
Linear Survey-Wave Trend	0.967 (0.914–1.024) p=0.248	0.964 (0.897–1.037) p=0.324
Sports × Linear Survey Wave	—	1.005 (0.927–1.090) p=0.896
Alcohol Use (Ref: No)	2.169*** (1.959–2.401) p<0.001	2.169*** (1.959–2.401) p<0.001
Other Illicit Drug Use (Ref: No)	3.691*** (3.347–4.069) p<0.001	3.691*** (3.347–4.069) p<0.001
Male (Ref: Female)	0.758*** (0.696–0.824) p<0.001	0.758*** (0.696–0.824) p<0.001
	Grade (Ref: 9th)	
10th Grade	0.932 (0.831–1.047) p=0.233	0.932 (0.830–1.047) p=0.233
11th Grade	0.870* (0.771–0.982) p=0.024	0.870* (0.771–0.982) p=0.024
12th Grade	0.741*** (0.652–0.841) p<0.001	0.741*** (0.652–0.841) p<0.001
	Race/Ethnicity (Ref: White)	
American Indian/Alaska Native	1.442 (0.945–2.200) p=0.089	1.442 (0.945–2.200) p=0.089
Asian	1.150 (0.923–1.434) p=0.211	1.151 (0.923–1.434) p=0.211
Black/African American	1.101 (0.928–1.305) p=0.268	1.101 (0.929–1.305) p=0.267
Hispanic/Latino (any)	1.234** (1.085–1.404) p=0.001	1.235** (1.085–1.404) p=0.001
Multiple Non-Hispanic	1.344** (1.108–1.632) p=0.003	1.344** (1.108–1.632) p=0.003
Native Hawaiian/Pacific Islander	1.028 (0.534–1.979) p=0.934	1.028 (0.534–1.979) p=0.934
Observations	40,182	40,182

Note. Results are aOR (95% CI), followed by p-value. Survey wave was coded sequentially from 0 to 3 for 2017, 2019, 2021, and 2023; trend aORs therefore represent change per two-year survey wave. Both models adjust for sports participation, alcohol use, other illicit drug use, sex, grade, and race/ethnicity. The interaction model adds sports participation × survey wave. aOR = adjusted odds ratio; CI = confidence interval. *** p<0.001; ** p<0.01; * p<0.05; — = not included in model.
